# Unexpected Myasthenic Crisis in Anesthesia for Magnetic Resonance Imaging – Diagnosis and Management

**DOI:** 10.7759/cureus.34959

**Published:** 2023-02-14

**Authors:** João Saldanha Marques, Patrícia Santos

**Affiliations:** 1 Anesthesiology, Centro Hospitalar Universitário de São João, Porto, PRT

**Keywords:** communication gap, organizational gaps, intraoperative complication, anesthesia at remote locations, patient safety, safety gaps, magnetic resonance imaging, myasthenia gravis crisis, myasthenia gravis (mg)

## Abstract

Myasthenic crisis (MC) is a rare and life-threatening manifestation of myasthenia gravis (MG) and is characterized by rapidly progressing weakness of respiratory and bulbar muscles leading to immobility, severe dyspnea, respiratory insufficiency, and possible aspiration. Early recognition and prompt treatment may prevent the development of further complications and the need for intubation, invasive mechanical ventilation, and ICU admission. Its diagnosis requires a high degree of clinical suspicion, and anesthesiologists, despite being prepared to deal with and provide anesthesia care to patients with MG, may not be immediately aware of the correct diagnosis and treatment of this medical emergency, and of the red flags that should prompt more invasive measures. Due to the very low incidence and possibly high morbidity of the condition, it is important to report cases of perioperative MC to raise awareness for early diagnosis and treatment. This case also emphasizes the importance of pre-anesthetic consultation and a multidisciplinary approach with appropriate communication and referral between medical specialties as the diagnosis of MG was not disclosed to the anesthetic team. The organizational, communication and safety gaps that occurred during the perioperative period could potentially have had a negative impact on patient outcomes. We report a case of MC in a patient who underwent general anesthesia for ambulatory magnetic resonance imaging and whose diagnosis of MG was not conveyed to the anesthesia care team.

## Introduction

Myasthenia gravis (MG) is a chronic autoimmune, neuromuscular disorder characterized by impaired transmission at the neuromuscular junction (NMJ). Despite its rarity, it is the most common autoimmune disorder affecting the NMJ, with an incidence rate of 4.1 to 30 cases per million [1]. Incidence rates vary with sex, showing a bimodal distribution in women with peaks at 30 and 50 years of age [1]. In men, the incidence increases steadily with age [1]. MG is caused by immunoglobulin G (IgG) autoantibodies working against the acetylcholine (ACh) receptor [1], detectable in the serum of 80-90% of affected patients. These autoantibodies are believed to play a central role in the disease process as they impair transmission at the NMJ by direct inhibition of ACh binding to the receptor and increased degradation of ACh receptors [1]. Clinically, this reflects in fatigable muscle weakness, which is the major symptom of MG, and is worsened by exercise and improves with rest [1]. This may affect extraocular muscles (resulting in ptosis and diplopia), bulbar muscles (causing dysarthria and pulmonary aspiration), and respiratory muscles. The mainstay of the treatment of MG is immunotherapy and symptomatic treatment with acetylcholinesterase inhibition [2]. There is also evidence that thymectomy is beneficial and improves symptoms in over 50% of patients [2,3].

Myasthenic crisis (MC) is a potentially life-threatening manifestation of MG and is defined as respiratory failure or delayed postoperative extubation for > 24h in a patient with MG due to weakness of the upper airway and respiratory muscles [4]. The overall prevalence is very low, as MG per se is rare. Despite this, 20% of patients with MG experience at least one episode of MC in their lives. Weakness may develop within minutes to days and is characterized by flaccid tetraparesis with immobility, severe dyspnea, respiratory insufficiency, and aspiration, which may require positive pressure ventilation, either non-invasive or invasive, and intensive care management [5]. Whether due to poor patient adherence to the therapeutic regimen or inadequate dosing, undertreatment is an important cause of MC. A precipitating factor can usually be identified in patients with myasthenic crises [4]. Thomas et al. retrospectively reviewed the hospital records of 53 patients admitted with a diagnosis of MC and reported that the most common identifiable triggers were infection (most often pneumonia or upper respiratory tract infection) and pulmonary aspiration [6]. Some crises may be precipitated by stress, surgical procedures, pregnancy, rapid tapering of immunomodulatory therapy, the beginning of the treatment with corticosteroids, and drugs that may worsen the symptoms of MG (e.g. macrolides, aminoglycosides, beta-blockers, calcium-channel blockers, lithium, muscle relaxants, magnesium, volatile anesthetic agents) [3,4].

We report a case of MC in a patient who underwent general anesthesia for ambulatory magnetic resonance imaging (MRI) and whose diagnosis of MG was not disclosed to the anesthesia care team during the pre-procedure assessment.

## Case presentation

We present the case of a 69-year-old woman (weight: 85 kg, height: 162 cm, body mass index: 32,4 kg/m^2^) who was admitted for an ambulatory abdominal MRI for the characterization of a polypoid lesion in the gastric antrum. In the bedside assessment performed immediately before the procedure, when asked about comorbidities and medications, she mentioned she had type 2 diabetes controlled with lifestyle interventions and depression treated with venlafaxine 75 mg/day. The patient was also on continuous positive airway pressure (CPAP) at night (pulmonology prescription) which we presumed to be for the treatment of obstructive sleep apnea. Regarding her previous anesthetic and surgical history, she mentioned an uneventful removal of benign tumors in the chest. She denied any allergies. Fasting times were carried out in accordance with the guidelines. Physical examination was unremarkable except for a mini-sternotomy scar. The patient was scheduled for a morning period usually reserved for pediatric MRI, which requires the presence of an anesthesiologist. The patient’s MRI procedure was initially supposed to be done without sedation/anesthesia, but upon arrival at the MRI admission area, anesthesia collaboration was requested because the patient had claustrophobia. 

In the MRI room, an MRI-compatible anesthesia workstation was available. The patient was monitored according to American Society of Anesthesiologists (ASA) standards, and general anesthesia was induced with 80 mg of propofol and maintained using a circle-absorber breathing circuit with sevoflurane (MAC 0.7-1.0) administered through a facemask. Spontaneous ventilation was maintained throughout the entire procedure. Vital signs were monitored from outside the MRI room with wireless MRI-compatible devices. At the end of the procedure, after sevoflurane discontinuation, the patient, despite being conscious, exhibited poor ventilatory dynamics, incapable of fully opening her eyes or even speaking, with the need for assisted ventilation with a bag-valve-mask device. MG and MC were suspected (strongly supported by past surgical history and clinical presentation). A further review of past medical history, including her daughter’s information, revealed a diagnosis of generalized MG, medicated with pyridostigmine, azathioprine, and methotrexate, and she was indeed on nocturnal bilevel positive airway pressure (BiPAP) ventilation. The patient was previously submitted to thymectomy and surgical correction of bilateral ptosis and was routinely followed at a neurology department of another hospital. In addition, the patient had chronic microcytic anemia due to a gastric ulcer and osteoarthritis. Arterial blood gas (ABG) analysis presented type 2 respiratory insufficiency (Table 1) (refer to column 1st ABG), but the patient had normal SpO_2_ with assisted ventilation with a bag-valve-mask device with 100% oxygen. A total of 2.5 mg of intravenous (IV) neostigmine (5 boluses of 0,5 mg) plus 200 mg of IV hydrocortisone were administered, with the patient being monitored. Immediate improvement was noticed after the administration of neostigmine, and full recovery occurred after 30 minutes, with adequate ventilatory dynamics and level of consciousness and improvement in blood gas parameters (Table 1) (refer to column 2nd ABG).

**Table 1:** Blood gas analysis results during diagnosis and management of myasthenic crisis ABG – arterial blood gas

Arterial blood gas parameter	1^st^ ABG (at myasthenic crisis presentation)	2^nd^ ABG (30 minutes after acute symptomatic and immunomodulatory treatment)	3^rd^ ABG (after resolution of respiratory acidosis, at the emergency department)	Reference values
pH	7.08	7.30	7.39	7.35-7.45
pCO_2_ (mmHg)	95	48.8	41.8	35.0-45.0
pO_2_ (mmHg)	136	344.1	95.2	75.0-105.0
HCO_3_^- ^(mmol/L)	27.8	23.2	25.3	22.0-28.0
Lactate (mmol/L)	1.19	1.20	0.78	0.0-2.0

**Figure 1:**
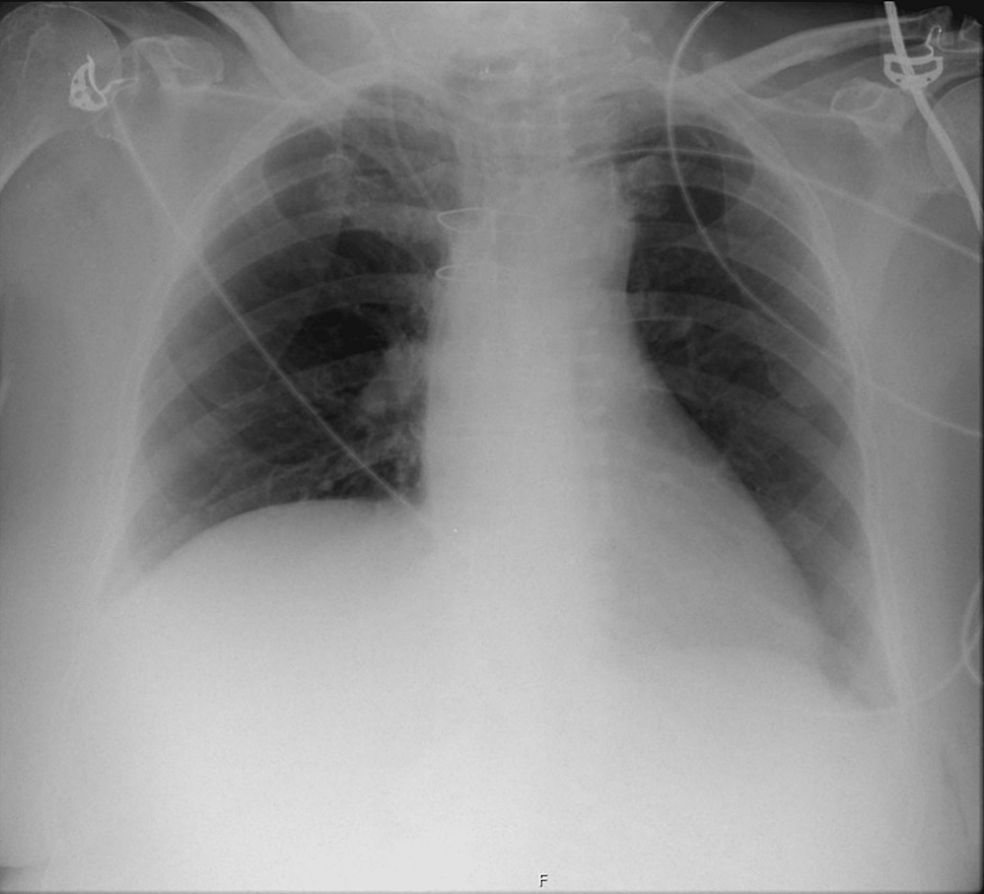
Chest x-ray at the emergency department

Oxygen therapy with a non-rebreathing mask with FiO_2_ 50% was applied, and the patient was transferred to the emergency department to be closely monitored and to initiate non-invasive ventilation (NIV). At admission to the emergency department, the patient was conscious but still somnolent. Respiratory dynamics were restored, and the patient was hemodynamically stable. The patient was assessed by neurology, which stated that this episode was probably related to the use of sevoflurane for maintenance of anesthesia during MRI and not to the progression of the disease. The neurological examination revealed horizontal diplopia with the levoversion and impaired abduction of the left eye. Oral pyridostigmine (60 mg) was then administered. ECG revealed a 1st-degree atrioventricular block and complete right bundle branch block (both present in previous ECG). The results of blood tests were normal except for a hemoglobin value of 6.5 g/dL (in the emergency department the patient mentioned she had had dark-colored stools in the last week which was consistent with the diagnosis of melena). Due to low hemoglobin value, one unit of packed red cells was administered. A chest x-ray was performed approximately one hour and 30 minutes after the presentation of MC (Figure 1). The x-ray showed no signs of pulmonary aspiration. Nevertheless, pulmonary aspiration might not be immediately apparent after the episode and unless there is a gross aspiration with subsequent atelectasis, there will be no acute changes in the chest x-ray. 

After the resolution of respiratory acidosis (Table 1) (refer to column 3rd ABG), NIV was exchanged to a nasal cannula with O_2_ 1L/min and the patient maintained hemodynamic stability and adequate respiratory dynamics throughout the rest of her stay at the emergency department. After eight hours of close monitoring in the emergency department, the patient was discharged home the same day, without further complications.

## Discussion

Despite being prepared to deal with and provide anesthesia care to patients with MG, anesthesiologists may not be sufficiently aware of the correct diagnosis and treatment of MC, as it is an infrequent perioperative as well as critical care complication. Early diagnosis and treatment of MC are essential and can avoid the need for invasive mechanical ventilation (IMV). In this regard, clinical examination and medical history are vital.

There is some controversy regarding the use of acetylcholinesterase inhibitors during the management of MC, especially if given intravenously, as they can increase respiratory secretions, complicate airway management, and have been associated with fatal cardiac dysrhythmia [2,7]. Despite it, some authors advocate the use of pyridostigmine or neostigmine for acute symptomatic therapy in addition to plasma exchange (PE)/immunoglobulin (IG) for acute treatment of the cause as well as the beginning or intensification of immunomodulatory therapy (cortisone, azathioprine, or rituximab) [5]. We decided to use intravenous neostigmine for acute symptomatic treatment of MC as it was the only acetylcholinesterase inhibitor immediately available in our anesthesia cart. In addition, a bolus of corticosteroid was administered as part of immunomodulatory therapy. The medications were administered under close monitoring, and the patient showed good clinical response to them. Due to bulbar symptoms possibly leading to pulmonary aspiration and/or respiratory insufficiency, early intubation might be needed to secure the airway [5]. NIV could be a helpful tool in preventing intubation [5,7,8] and was used as a bridging therapy, both during the physical examination and while the medical history was obtained as well as throughout the treatment. Neumann et al. showed that the application of NIV appeared to be safe in MC and was sufficient in 38% of cases of MC, especially if combined with early treatment with PE or immunoadsorption, so that intubation and IMV become unnecessary, resulting in a shorter length of the crisis and ICU stay [8]. Once the patient is mechanically ventilated, the duration of ventilation is typically five to seven days. Early extubation after only a few days of IMV often results in reintubation, so a conservative approach regarding extubation may be recommended [2].

Although undertreatment is an important cause of MC, some drugs that may increase myasthenic weakness and trigger an MC episode, such as sevoflurane, despite not being contraindicated, should be used with caution as it can exert an inhibitory effect on neuromuscular transmission [9]. If no previous medical record was withheld and if formal pre-operative checks were conducted, we would have probably chosen another anesthetic technique, avoiding the use of volatile anesthetic agents. Total intravenous anesthesia (TIVA) may be the preferred approach in patients with MG. Dexmedetomidine could have a role in cases requiring sedation as it also preserves spontaneous ventilation without enhancing myasthenic weakness. Anemia could have also played a minor role in the MC episode as it affects the oxygen-carrying capacity and may exacerbate even further muscle weakness [9].

This case also reiterates the importance of a pre-anesthetic consultation before the procedure with a review and optimization of the patient’s diseases and medications and checking past anesthetic charts and surgical records, especially in high-risk cases/patients. A multidisciplinary approach would have been more suitable in this case with appropriate communication and referral between medical specialties, as many of the organizational, communication and safety gaps that occurred during the peri-procedure period could potentially have had a negative impact on patient outcomes. Failures in preparation, teamwork, and communication can contribute significantly to patient harm [10]. In this case, the best way to proceed would probably involve an early referral to a pre-anesthetic consultation, as the patient required the procedure to be performed under sedation/anesthesia and to schedule the MRI on an in-patient basis.

## Conclusions

Despite the occurrence of an MC, early identification of the pathologic process and prompt treatment prevented the development of further complications and the need for invasive mechanical ventilation and admission to the ICU. A multidisciplinary approach involving anesthesiologists, emergency doctors, and neurologists allowed for the appropriate management of the crisis and resulted in a favorable outcome. The organizational, communication, and safety gaps presented to stress the need for effective preparation, teamwork, and communication, as it can decrease the incidence of perioperative incidents and improve patient safety.
